# An avoidable cause of thymoglobulin anaphylaxis

**DOI:** 10.1186/s13223-017-0186-9

**Published:** 2017-02-23

**Authors:** S. Brabant, A. Facon, F. Provôt, M. Labalette, B. Wallaert, C. Chenivesse

**Affiliations:** 1 Department of Allergy-Immunology, Pole de Biologie Pathologie Genetique Medicale du CHRU de Lille, Boulevard du professeur Leclercq, 59037 Lille Cedex, France; 2 Department of Anaesthesiology, CHU Lille, 59000 Lille, France; 3 Department of Kidney Transplantation, CHU Lille, 59000 Lille, France; 4 Department of Pulmonology, Allergy and Immunology, CHU Lille, 59000 Lille, France

**Keywords:** Thymoglobulin, Anaphylactic allergic reaction, Rabbit proteins

## Abstract

**Background:**

Thymoglobulin® (anti-thymocyte globulin [rabbit]) is a purified pasteurised, gamma immune globulin obtained by immunisation of rabbits with human thymocytes. Anaphylactic allergic reactions to a first injection of thymoglobulin are rare.

**Case presentation:**

We report a case of serious anaphylactic reaction occurring after a first intraoperative injection of thymoglobulin during renal transplantation in a patient with undiagnosed respiratory allergy to rabbit allergens.

**Conclusions:**

This case report reinforces the importance of identifying rabbit allergy by a simple combination of clinical interview followed by confirmatory skin testing or blood tests of all patients prior to injection of thymoglobulin, which is formally contraindicated in patients with a history of hypersensitivity to rabbit proteins.

## Background

Thymoglobulin is an IgG fraction purified from the serum of rabbits immunised against human thymocytes. The preparation consists of polyclonal antilymphocyte IgG directed against T lymphocyte surface antigens, and induction of profound lymphocyte depletion is though to be the main mechanism of thymoglobulin-mediated immunosuppression. Thymoglobulin is now part of the standard treatment for intraoperative induction of immunosuppression during renal transplantation [1, 2]. It can also be used for the prevention and treatment of graft-versus-host reaction during haematopoietic stem cell transplantation and other organ transplantation, and for the treatment of bone marrow aplasia [3–6].

The dosage of thymoglobulin depends on the indication, the treatment regimen and the possibility of use in combination with other immunosuppressive drugs. Infusion must be performed under strict surveillance in hospital. The main contraindication to administration of thymoglobulin is hypersensitivity to rabbit proteins [7]. Cases of serious anaphylactic reactions to thymoglobulin due to rabbit protein allergy are very rare, and consequently, specific tests for rabbit allergy are not usually performed as part of the pre-transplant assessment. We report a case of serious anaphylactic reaction due to rabbit protein allergy following a first injection of thymoglobulin.

## Case presentation

A 24-year-old Caucasian man was hospitalised in March 2015 for renal transplantation. He had a history of spina bifida, neurogenic bladder with Bricker ileal conduit complicated by chronic renal failure requiring haemodialysis since 2011; and atopy, comprising respiratory allergy to pollens and house dust mites (asthma and rhinitis), contact allergy to latex (angioedema following inflation of a toy balloon), and vancomycin drug allergy.

The patient was undergoing renal transplantation for the first time. Surgery was performed in a latex-free operating room. General anaesthesia was induced by intravenous (IV) administration of propofol (150 mg IV push), ketamine (35 mg IV push) and sufentanil (20 µg IV push). Tracheal intubation was performed without complication and the patient received antibiotic prophylaxis by cefoxitin (2 g IV). Forty-five minutes after induction, thymoglobulin immunosuppression (12.5 mg/h of 5 mg/mL in 50 mL 5% dextrose to be administered over 8 h) was initiated. Within 3 min of the start of the thymoglobulin infusion, the patient experienced hypotension of 56/35 mmHg and extreme bradycardia (43 bpm) secondary to hypoxia, requiring resuscitation with external cardiac massage (for less than 1 min) and administration of adrenaline (0.1 mg IV push) (Table 1). The thymoglobulin infusion was stopped immediately (at which point 0.625 mg had been infused). The patient concomitantly presented with generalised erythroderma and bronchospasm. He was subsequently managed in the intensive care unit and the operation was deferred. A favourable course was observed in response to adrenaline 2 mg/h, despite another episode of bronchospastic respiratory distress several hours later than required terbutaline nebulisation. A tryptase assay performed 1 h after onset of anaphylactic shock was elevated to 182 µg/L (normal 10.5 µg/L) confirming the anaphylactic origin of the shock. Histamine was also elevated to 234 nmol/L (normal < 10 nmol/L).

**Table 1:** Hemodynamic parameters during the operative period

	Before general anaesthesia	Induction	Before infusion of ATG	3 min post-infusion of ATG	5 min post-infusion of ATG
Systolic bp (mmHg)	179	143	123	56	Cardiac asystole External cardiac massage Adrenaline Thymoglobulin infusion stopped
MAP (mmHg)	139	111	96	43	
Diastolic bp (mmHg)	121	89	76	35	
HR (bpm)	100	98	72	124	
PaCO_2_ (mmHg)	Not intubated	39	32	24	
SpO_2_ (%)	94	98	99	95	

*ATG* thymoglobulin, *bp* blood pressure, *HR* heart rate, *MAP* mean arterial pressure, *PaCO*_*2*_ partial pressure of CO_2_, *SpO*_*2*_ haemoglobin oxygen saturation

To explore the origin of the shock, the patient was hospitalised for allergy testing in September 2015. A clinical interview retrospectively revealed a history of atopy with asthma and allergic rhinitis and contact hypersensitivity to rabbits triggering asthma and facial oedema. Skin tests were performed. Positive (histamine dihydrochloride 10 mg/mL equivalent to 1% or 5.43 mmol/L, Stallergenes, France) and negative (glycerinate solution) controls were included. Skin prick tests (SPTs) were carried out and interpreted according to international guidelines [8]. The results were read after 10 min and the largest diameter was recorded. A skin reaction of ≥3 mm was considered positive. The histamine positive control response was small (wheal of 2 mm). SPTs and intradermal tests (using a sterile solution of the suspected drug serially diluted in phenolated saline) were negative for all medications used during induction of anaesthesia [9]: sufentanil, propofol, and ketamine at 10^−3^, 10^−2^, and 10^−1^ dilutions; suxamethonium, rocuronium bromide, and cisatracurium besylate at 10^−3^ and 10^−2^ dilutions; and atracurium besylate and mivacurium chloride at 10^−1^ dilution. An intradermal test for colloid (Voluven®) was negative at 10^−3^, 10^−2^, and 10^−1^ dilutions. In contrast, a SPT for thymoglobulin was positive, with an 8 mm wheal at a dilution of 10^−6^. Additional SPTs were performed using commercial standardised extracts and prickers (Alyostal Prick®, Stallergenes, France). The panel included the following allergenic extracts at 100 IR/mL or 100 IC/mL: rabbit, hamster, guinea pig, and horse epithelia, and latex (a known allergen for this patient). The SPTs for rabbit and horse epithelia were positive at 5 mm; the test for latex was also “positive” but was only 2 mm. A blood sample was taken for measurement of specific IgE (ImmunoCap system, ThermoFisher, Uppsala, Sweden). IgE for quaternary ammonium and suxamethonium were <0.10 kU/L and specific IgE for penicillin and cefaclor were negative. Specific IgE to rabbit dander and rabbit serum proteins were elevated at 10.2 and 2.22 kU/L, respectively. IgE specific to rabbit meat was 0.13 kU/L.

On the basis of clinical and laboratory data, we concluded that the patient had experienced a grade IV anaphylactic reaction to thymoglobulin due to rabbit protein allergy. All medications containing rabbit proteins will be permanently contraindicated for this patient and an allergy card was issued. To date, the patient has not been transplanted.

## Discussion

Anaphylaxis is a hypersensitivity reaction that can rapidly become life threatening.

Thymoglobulin administration is generally uneventful and anaphylactic reactions are rare. Only 3 cases of allergic anaphylaxis to thymoglobulin have been reported in the literature [10–12]; one was a fatal reaction to a thymoglobulin carbohydrate excipient rather than anti-thymocyte globulin itself [11]. In the two other cases, renal transplant recipients experienced severe IgE reactions to thymoglobulin, similar to our case report, but skin tests and tryptase dosage were not performed in one case [10] and, in the third case, tryptase level was markedly increased but no skin test was performed to confirm anaphylaxis to thymoglobulin [12]. Reactions to thymoglobulin are usually due to release of cytokines by activated monocytes and lymphocytes, known as severe cytokine release syndrome, which can be associated with serious cardiovascular events. The most common complications are haematological (thrombocytopenia, granulocytopenia). However, rare cases of serious complications following thymoglobulin administration have been reported, including serum sickness [13], cardiopulmonary failure [14] and acute respiratory distress syndrome [15].

The case of anaphylactic shock reported here was observed within minutes of thymoglobulin administration, at which time only a very small dose had been infused (less than 1 mg). The infusion was immediately stopped. This serious reaction required management in the intensive care unit and postponement of transplantation. The rapid onset and severity of the anaphylactic reaction argue in favour of IgE-mediated anaphylaxis. The patient had positive SPTs to both thymoglobulin and rabbit dander, strongly implicating a rabbit protein allergic mechanism. The weak positivity of histamine SPT was probably related to end stage kidney failure.

Reports of anaphylactic reactions to rabbit proteins are rare [16, 17]. One case occured after minor injury with a needle that had been used on rabbit tissue [17]. The patient had a history of respiratory allergy to rabbits, suggesting a similar mechanism to that reported here. Rabbits are becoming increasingly popular as domestic pets and lead to sensitization in 1.5% of rabbit ownerships [18]. Although rare, one cannot excluse the possibility of cross sensitization between domestic pet [19]. The significant prevalence of rabbit sensitization and the severity of allergic reactions related to intravenous infusion of rabbit antibodies support the attitude that consists in investigating systematically rabbit sensitization before the use of preparations containing rabbit antibodies. However, screening for rabbit protein allergy is rarely performed in the context of complex pre-transplant assessment, despite the frequent use of thymoglobulin immunosuppression. We think that standardized evaluation including skin prick-tests to rabbit epithelia should be systematically performed before rabbit antibodies infusion. Millar et al. suggested that skin-testing to thymoglobulin should be performed systematically before anti-thymocyte globulin injection to aid in determining the hypersensitivity status [20]. However, the use of anti-thymocyte globulin for skin-testing is limited by its availability and price.

## Conclusion

Diagnosis of rabbit protein allergy is the only way to prevent thymoglobulin anaphylaxis such as the case reported here. This can be accomplished very easily through a simple combination of clinical interview followed by confirmatory SPT or blood tests.

## Abbreviations

ATG: anti-thymocyte globulin (thymoglobulin)
Ig: immunoglobulin
IC: concentration index
IR: index of reactivity
IV: intravenous
SPT: skin prick test
